# Inverse Gas Chromatography for Characterization of Adsorption Ability of Carbon–Mineral Composites for Removal of Antibiotics from Water

**DOI:** 10.3390/ma19020419

**Published:** 2026-01-21

**Authors:** Piotr Słomkiewicz, Katarzyna Piekacz, Sabina Dołęgowska

**Affiliations:** Institute of Chemistry, Jan Kochanowski University, 25-406 Kielce, Poland; katarzyna.piekacz@interia.pl (K.P.); sabina.dolegowska@ujk.edu.pl (S.D.)

**Keywords:** antibiotics, carbon–mineral composites, removal efficiency, IGC

## Abstract

In this study, inverse gas chromatography (IGC) was applied to characterize the key surface physicochemical properties of carbon–mineral composites and to clarify how these properties relate to removal efficiencies of selected antibiotics, with particular emphasis on surface energetic and acid–base characteristics rather than bulk structural parameters. The dispersive component of surface free energy and the acid–base characteristics (*K_a_*/*K_b_* ratio) were determined, alongside measurements of carbon content, while specific surface areas were compared with data reported previously. We found that there is no clear correlation between bulk structural characteristics and the removal efficiency of ciprofloxacin, doxycycline, sulfamethoxazole, and tetracycline. In contrast, the removal of all investigated antibiotics was found to be correlated with the dispersive component of surface free energy and the *K_a_*/*K_b_* ratio. The results suggest that surface energetic parameters and acid–base properties are more closely associated with antibiotic adsorption behavior than basic structural characteristics alone. These findings demonstrate that IGC provides valuable insight into adsorption processes and highlight the importance of surface physicochemical properties for interpreting and predicting the adsorption properties of carbon–mineral composites.

## 1. Introduction

Antibiotics have been recognized as a new class of emerging contaminants (micropollutants) that have been detected worldwide in various environmental matrices, including surface water, groundwater, soil, and sediment. In the environment, antibiotics may occur in parent form or as bioactive metabolites, affecting aquatic and terrestrial organisms even at low concentrations. They may also alter the structure and function of microbial communities and disturb nutrient cycles. One of the most serious concerns is the development and spread of antibiotic-resistant organisms, which leads to a gradual decrease in the effectiveness of clinically important antibiotics. The widespread occurrence of antibiotics is largely due to their incomplete removal by conventional wastewater treatment processes, including activated sludge and standard biological, chemical, and physical processes [1,2,3,4,5,6,7,8,9].

Various methods have been proposed for removing antibiotics from water, such as membrane filtration, advanced oxidation processes, algae-based treatments, and hybrid technologies. Increasing interest has been directed towards novel carbon-based materials, such as carbon–mineral composites. These composites can be produced from a variety of materials, including organic waste and minerals. This reduces processing costs and enables waste streams to be reused, in line with the principles of the circular economy [10,11,12,13,14,15]. However, the effective application of such materials requires well-defined composite preparation procedures, comprehensive structural characterization to allow prediction of their adsorption properties, and an understanding of which parameters actually drive adsorption processes.

In our previous studies, we took adsorption measurements of selected antibiotics: ciprofloxacin, doxycycline, sulfamethoxazole, and tetracycline were reported on raw halloysite minerals, halloysite nanotubes, kaolinite, and carbon–mineral composites [16,17,18]. These compounds are extensively used to treat a broad range of infections and are therefore frequently detected in the environment. A brief characterization of the investigated antibiotics and their environmental relevance is compiled in Table 1. The carbon–mineral composites were obtained by carbonization under a nitrogen atmosphere, using halloysite and kaolinite as mineral carriers and fruit pomace as the carbon precursor, and characterized using several methods [16,17,18]. However, the structural parameters obtained did not fully account for the observed differences in antibiotic removal. This suggests that a more complete interpretation requires evaluation of adsorption thermodynamics. Accordingly, in the present work, we determine the thermodynamic functions of adsorption for all tested materials.

Considering this, in the present study, raw minerals and carbon–mineral composites were characterized by inverse gas chromatography (IGC). We examined how adsorption thermodynamic parameters (surface free energy) and acid–base properties, determined by ICG, influence the adsorption of ciprofloxacin, doxycycline, sulfamethoxazole, and tetracycline onto these materials. In addition, the carbon content of the carbon–mineral composites was determined, and the specific surface areas were compared with values reported in our previous studies [16,17,18]. A better understanding of the impact of these parameters on the adsorption process can help to explain the adsorption abilities of mineral and carbon–mineral composites.

## 2. Background Information

The synthesis conditions of carbon–mineral adsorbents are summarized in Table 2. The porous structure parameters of the obtained composites were determined using low-temperature nitrogen adsorption isotherms (−196 °C), measured with a volumetric adsorption analyzer (ASAP 2020, Micromeritics, Norcross, GA, USA). Basic structural parameters of the obtained composites are summarized in Table 3.

A summary of the removal efficiencies of ciprofloxacin, doxycycline, sulfamethoxazole, and tetracycline on raw halloysite minerals (HSs), halloysite nanotubes (HNTs), kaolinite (KT), and carbon–mineral composites, determined in batch experiments at 25 °C, is presented in Table 4 [16,17,18]. In all cases, the initial pH of the antibiotic solution resulted in the antibiotic being predominantly in a neutral and/or zwitterionic form, which is typically associated with the highest adsorption affinity.

The results indicate that there is no clear correlation between the structural parameters (Table 3) and the efficiencies of antibiotic removal (Table 4). Therefore, it is not possible to establish a direct relationship between these quantities, nor demonstrate that the degree of antibiotic removal depends on the adsorbent structure. To address this limitation, inverse gas chromatography (IGC) could be used. IGC is a versatile and powerful technique for characterizing the physicochemical properties of adsorbents. In the adsorption process, two main types of interaction can be distinguished between the adsorbent surface and the adsorbate in the adsorption process, namely, London dispersion interactions (interactions between instantaneous dipoles and induced dipoles) and specific interactions (resulting from the interactions of polarized functional groups). These interactions can be quantified through the dispersive component of the surface free energy. Additionally, adsorption depends on the acid–base properties of the adsorbent surface, which can be acidic (electron-accepting), basic (electron-donating), or amphoteric. The IGC method can be used to determine both the dispersive component of surface free energy and the acid–base properties of materials, providing valuable insight into adsorption processes [23].

Determination of the solid–vapor surface free energy of adsorption using the Schultz [24,25] and Dorris–Gray approaches [26,27] has been extensively described in the literature related to IGC methods. Therefore, Table 5 provides a concise summary of their theoretical assumptions and the method used to calculate the acid/base properties of the adsorbent surface.

## 3. Materials and Methods

### 3.1. Characterization of Adsorbents by the IGC Method

Chromatographic measurements were performed using the IGC SEA—Inverse Gas Chromatography and Surface Energy Analyzer (Surface Measurement Systems, Ltd. Alperton, United Kingdom)—equipped with a flame ionization detector. Helium was used as a carrier gas at a constant flow rate of 20 cm^3^·min^−1^ through the column. Retention measurements were carried out at 140 °C, while the detector and dispenser temperatures were maintained at 180 °C. Glass columns measuring 30 cm in length and 4 mm in internal diameter were used. Each adsorbent bed was isolated on both sides by approximately 2 cm of glass wool. The following materials were used as the stationary phase: HS, HNT, KT, CHS1a, CHNT1a, CKT1a, CHS1b, CHNT1b, and CKT1b (Table 2). The mass of the adsorbents ranged from 18 to 71 mg.

Test substances were divided into two groups: non-polar substances (hexane, heptane, octane, nonane) and polar substances (acetone, acetonitrile, ethyl acetate, dichloromethane). Methane was used as the calibration substance. The test substances were dosed onto the columns filled with the proper adsorbent using an autosampler. The surface coverage of the adsorbents ranged from 0.0063 to 0.7. Adsorption parameters of the adsorbents were calculated using the Cirrus Plus operational program.

### 3.2. Carbon Content Determination of Carbon–Mineral Composites

To determine the carbon content, the crucibles were first calcined at 800 °C to a constant mass. The tested adsorbents were dried for 24 h at 105 °C to remove moisture. Next, 1 g of each adsorbent was weighed in a pre-calcinated crucible and placed in a furnace preheated to 800 °C. The samples were heated for 90 min at this temperature, removed from the furnace, cooled to room temperature, and weighed. They were then re-calcined at 800 °C for an additional 15 min. The process of repeated roasting was continued until a constant mass was achieved (∆m = 0.001 g). The carbon concentrations determined in the carbon–mineral adsorbent samples are presented in Table 6.

## 4. Results and Discussion

The measured values of the dispersive component of the surface free energy and the acidic and basic characteristics of the minerals HS, HNT, and KT, as well as the carbon–mineral composites CHS1a, CHNT1a, CKT1a, CHS1b, CHNT1b, and CKT1b, are summarized in Table 7.

The specific surface areas of HS, HNT, KT, and the carbon–mineral composites ranged from 8.93 m^2^·g^−1^ to 77.01 m^2^·g^−1^ in the following order: KT < CKT1b < CKT1a < HS < HNT < CHS1b < CHS1a < CHNT1b < CHNT1a (Table 3). The carbon content of the carbon–mineral composites ranged from 76.1% to 85.7% in the increasing order CHNT1b < CHS1b < CKT1b < CHS1a < CHNT1a < CKT1a (Table 6), and was relatively similar across the samples. The degree of ciprofloxacin removal by mineral and carbon–mineral composites ranged from 25.1% to 67.5% in the direction CKT1a < CKT1b < HNT < KT < CHS1b < CHNT1b < HS < CHNT1a < CHS1a. The initial pH of the antibiotic solution was 6.5, at which ciprofloxacin was present in its zwitterionic form (Table 4). This speciation is generally favorable for adsorption, as it promotes hydrophobic and π–π interactions with the adsorbent surface [28].

**Figure 1:**
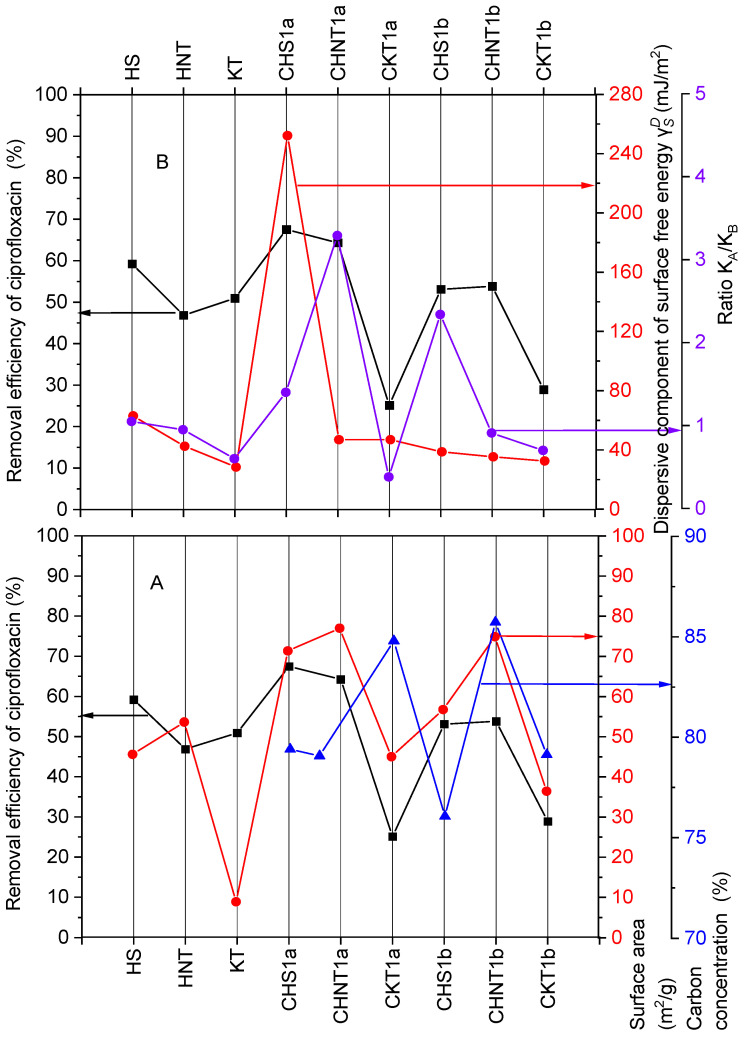
Dependence of ciprofloxacin removal efficiency by mineral and carbon–mineral composites on (**A**) specific surface area and carbon content after pyrolysis, and (**B**) $\frac{K_{a}}{K_{b}}$ ratio and dispersive component of the surface free energy $\gamma_{S}^{D}$.

The dependence of the ciprofloxacin removal efficiency on specific surface area cannot be determined based on these two parameters (Figure 1A), as the observed trend is ambiguous. For example, the removal efficiency was higher for CHS1a (67.5%) than for CHNT1a (64.3%), despite the fact that the specific surface area of CHS1a (71.38 m^2^·g^−1^) was lower than that of CHNT1a (77.01 m^2^·g^−1^). As the carbon contents of CHS1a and CHNT1a are similar, they probably do not significantly contribute to this difference. Instead, the explanation may lie in differences in thermodynamic surface properties, particularly the surface free energy and the *K_a_*/*K_b_* ratio. These properties describe the thermodynamic strength of adsorption, and the acid–base characteristics that contribute to the driving force [29].

The values of the dispersive component of the surface free energy of HS, HNT, and KT, as well as of the carbon–mineral composites, ranged from 28.4 mJ·m^−2^ to 252 mJ·m^−2^ in the following order: KT < CKT1b < CHNT1b < CHS1b < HNT < CHNT1a < CKT1a < HS < CHS1a (Table 7). Meanwhile, the ratio of the acidic to basic characteristics of the solid surface *K_a_*/*K_b_* varied in the range from 0.38 to 3.59 and increased in the sequence: CKT1a < KT < CKT1b < CHNT1b < HNT < HS < CHS1a < CHS1b < CHNT1a (Table 7).

Comparison of ciprofloxacin removal efficiencies for the mineral and carbon–mineral composites (Table 4) with the dispersive component of surface free energy and the *K_a_*/*K_b_* ratio (Figure 1B) indicates that the highest removal efficiency (67.5%) observed for CHS1a corresponds to the highest surface free energy value (252 mJ·m^−2^). Similarly, materials exhibiting relatively high *K_a_*/*K_b_* ratios, CHNT1a (3.29), and CHS1b (2.34), showed ciprofloxacin removal efficiencies of 64.3% and 53.1%, respectively. These observations suggest that the dispersive component of surface free energy and the *K_a_*/*K_b_* ratio play an important role in ciprofloxacin adsorption, rather than bulk structural parameters alone.

The degree of doxycycline removal by the mineral and carbon–mineral composites ranged from 36.7% to 95.7% in the order KT < CKT1b < CKT1a < HS < CHNT1b < HNT < CHNT1a < CHS1b < CHS1a. The initial pH of the antibiotic solution was 4.3, at which doxycycline was predominantly present in its zwitterionic form (Table 4). Under these conditions, adsorption may be promoted by π–π electron donor–acceptor interactions between the aromatic rings of doxycycline and the carbon phase, as well as by hydrogen bonding involving phenolic, amide, and hydroxyl groups [30,31].

**Figure 2:**
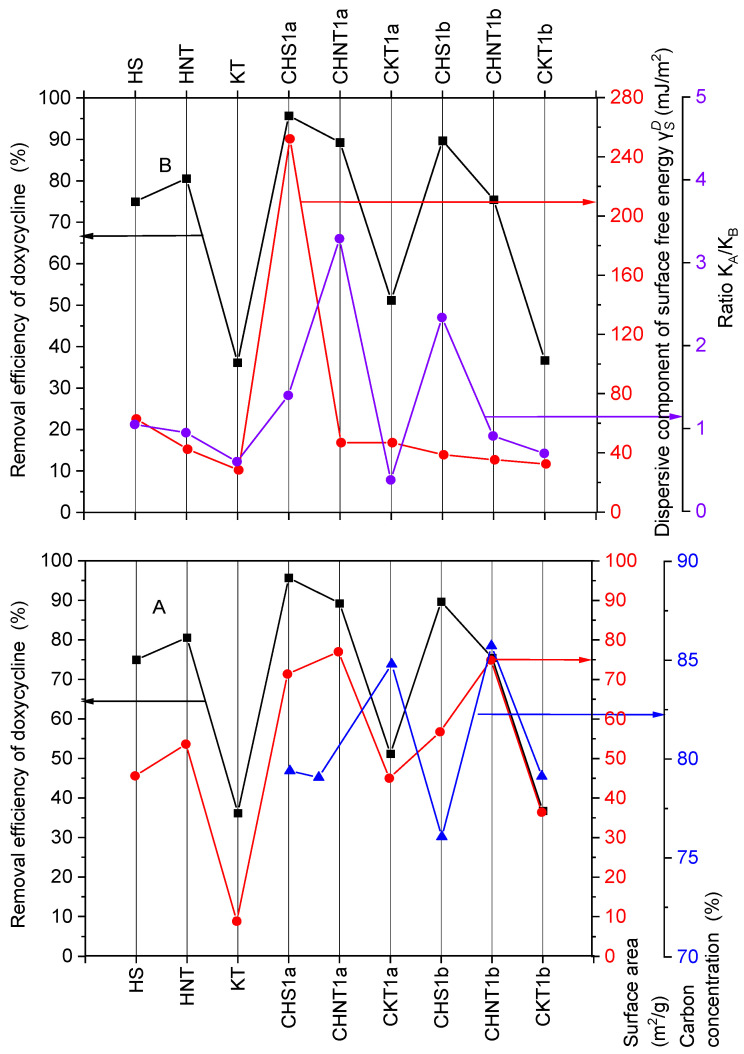
Dependence of doxycycline removal efficiency by mineral and carbon–mineral composites on (**A**) specific surface area and carbon content after pyrolysis, (**B**) $\frac{K_{a}}{K_{b}}$ ratio and values of components of surface free energy $\gamma_{S}^{D}$.

The dependence of doxycycline removal on specific surface area (Figure 2A) is also ambiguous. For example, CHS1a had a higher removal efficiency (95.7%) than CHNT1a (89.3%), although its specific surface area (71.38 m^2^·g^−1^) was lower than the specific surface area of CHNT1a (77.01 m^2^·g^−1^). The carbon content of CHS1a and CHNT1a is similar, so it is unlikely to have a significant impact on this difference.

The removal efficiency of doxycycline (95.7%) corresponds to the surface free energy component of the CHS1a composite (252 mJ·m^−2^) (Figure 2B). It can also be observed that the high *K_a_*/*K_b_* ratios for CHNT1a (3.29) and CHS1b (2.34) are associated with increased doxycycline removal efficiencies of 89.3% and 89.7%, respectively, compared to the results obtained for the other adsorbents.

The degree of sulfometaxazole removal by the mineral and carbon–mineral composites ranged from 4.25% to 65.4% in the order HS < KT < CHS1 < HNT < CKT1b < CHNT1b < CKT1a < CHNT1a < CHS1a (Table 4). The degree of sulfamethoxazole removal by the mineral and carbon–mineral composites ranged from 4.25% to 65.4% in the following order: HS < KT < CHS1b < HNT < CKT1b < CHNT1b < CKT1a < CHNT1a < CHS1a. The initial pH of the solution was 5.5, at which sulfamethoxazole was predominantly present in its neutral form (Table 4). Adsorption of sulfamethoxazole depends strongly on its neutral fraction, which favors the formation of hydrophobic interactions, π–π interactions, and hydrogen bonding. Its adsorption decreases when sulfamethoxazole is predominantly in cationic or anionic form [32,33].

**Figure 3:**
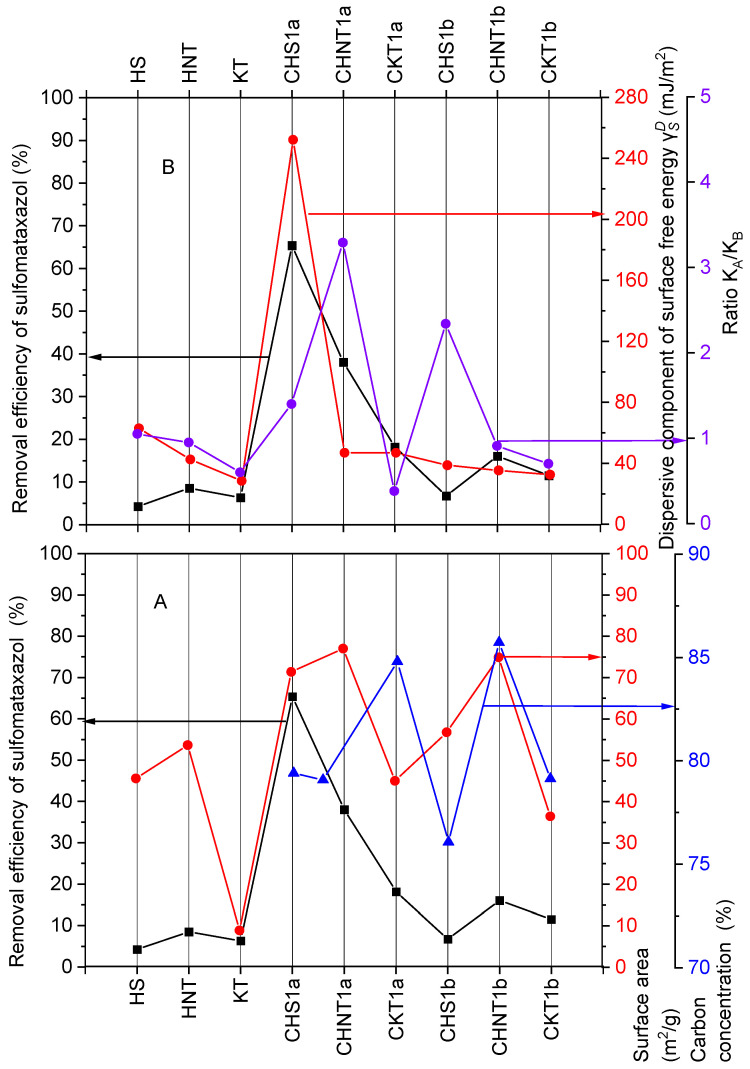
Dependence of sulfometaxazole removal efficiency by mineral and carbon–mineral composites on (**A**) specific surface area and carbon content after pyrolysis, (**B**) $\frac{K_{a}}{K_{b}}$ ratio and values of components of surface free energy $\gamma_{S}^{D}$.

For the materials HS, KT, CHS1, HNT, CKT1b, CHNT1b, and CKT1a, the degree of sulfometaxazole removal does not exceed 20% (Figure 3A). A contradictory relationship is also observed: for CHS1a the degree of sulfometaxazole removal was 65.4% at the specific surface area of 71.38 m^2^·g^−1^, whereas for CHNT1a the degree of removal was 38.0% at a higher specific surface area of 77.01 m^2^·g^−1^, despite similar carbon contents (Table 6).

The influence of specific surface area and carbon content on sulfometaxazole removal cannot be determined for the other adsorbents. For CHS1a only, there is a dependence of sulfometaxazole removal on the component of surface free energy and the *K_a_*/*K_b_* ratio (Figure 3B). For this composite, a degree of removal of 65.4% corresponds to a value of the component of surface free energy of 252 mJ·m^−2^. In all other cases, the curves representing these relationships are random.

For the last antibiotic, tetracycline, the removal efficiency ranged from 36.2% to 96.5% in the following order: CKT1b < KT < CKT1a < HS < CHS1b < HNT < CHNT1b < CHNT1a < CHS1a. The initial pH of the solution was 4.5, at which tetracycline was predominantly present in its zwitterionic form (Table 4). Tetracycline adsorption is typically highest when the zwitterionic or neutral species predominates and generally decreases at higher pH when tetracycline becomes anionic. These forms enable strong π–π interactions and hydrogen bonding [34,35].

**Figure 4:**
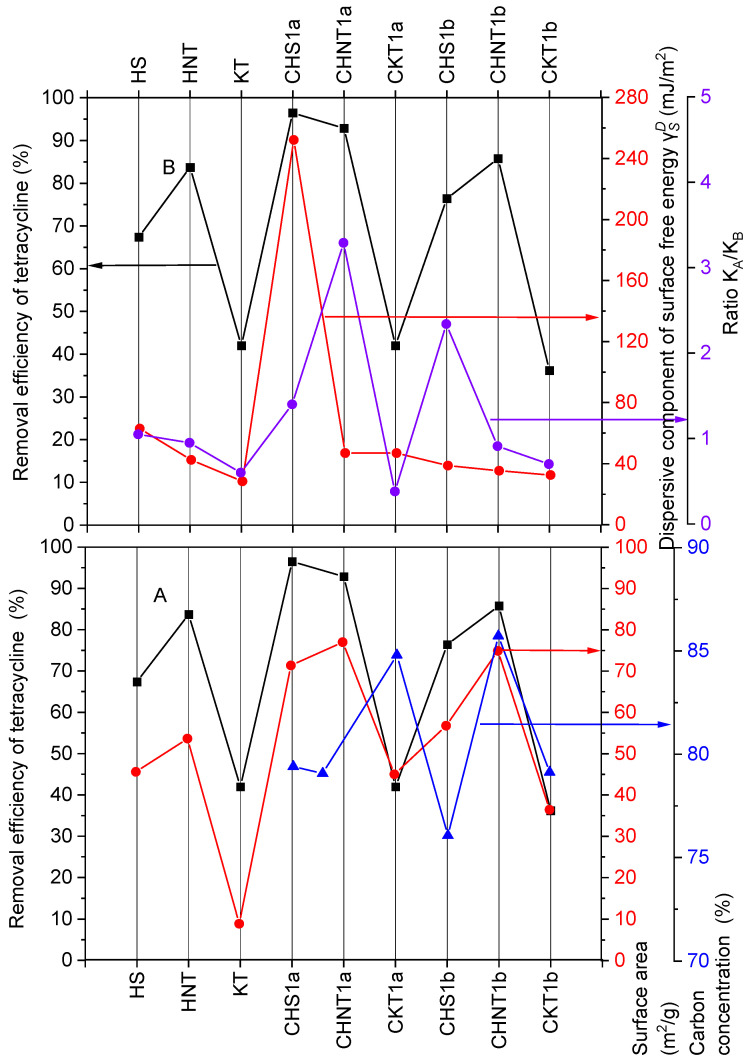
Dependence of tetracycline removal efficiency by mineral and mineral–carbon composites on (**A**) specific surface area and carbon content after pyrolysis, (**B**) $\frac{K_{a}}{K_{b}}$ ratio and values of components of surface free energy $\gamma_{S}^{D}$.

Figure 4A shows that a direct relationship was found between the degree of tetracycline removal by mineral and mineral–carbon composites, and the specific surface area for HS, HNT, CHS1b, and CKT1b. In two cases, CHS1a and CHNT1a, such a relationship was not observed. For CHS1a the removal efficiency was 96.5% and was higher than the removal efficiency for CHNT1a (92.9%). At the same time, the specific surface area of CHS1a was lower (71.38 m^2^·g^−1^) than the specific surface area of CHNT1a (77.01 m^2^·g^−1^).

This effect can be explained only by comparing the values of the dispersive component of the surface free energy, which was much higher for CHS1a (252 mJ·m^−2^) than for CHNT1a (46.8 mJ·m^−2^) (Figure 4B).

For the remaining adsorbents (HS, HNT, KT, CHNT1a, CHS1b, CHNT1b, and CKT1b), the values of the dispersive surface free energy were at a similar level and probably have a smaller impact on the degree of tetracycline removal. It can also be assumed that the carbon content does not significantly contribute to the tetracycline removal efficiency.

## 5. Conclusions

Previous studies on the adsorption of ciprofloxacin, doxycycline, sulfamethoxazole, and tetracycline on mineral and mineral–carbon composites focused on structural characterization of these adsorbents. These studies showed that establishing a clear relationship between antibiotic removal efficiencies and either carbon content or specific surface area is difficult.

Therefore, we used inverse gas chromatography to determine components of the surface free energy and donor–acceptor (acid–base) properties of mineral and mineral–carbon composite surfaces. To the best of our knowledge, no previous attempts have been made to relate the degree of antibiotic removal to the thermodynamic surface parameters of the adsorbents. The obtained parameters allowed us to explain differences in the adsorption of structurally and chemically distinct antibiotics (ciprofloxacin, doxycycline, sulfamethoxazole, and tetracycline) on the investigated materials.

The results confirmed that the surfaces of mineral and mineral–carbon composites have electron-acceptor properties. Moreover, the values of the dispersive component of surface free energy and the acid–base properties of HS, HNT, KT, and mineral–carbon composites showed that CHS1a has an extremely high surface energy compared with the other adsorbents, which is consistent with the highest removal efficiencies observed for all four antibiotics on CHS1a.

Overall, IGC enabled a more detailed interpretation of the adsorption process, linking the removal efficiencies of different antibiotics to the thermodynamic properties of the absorbent surfaces and the properties of antibiotics.

## Figures and Tables

**Table 1:** Physicochemical properties and environmental impact of antibiotics [14,16,17,18,19,20,21,22].

Antibiotic	Doxycycline	Tetracycline	Sulfametaxazole	Cyprofloxacin
**Structure and IUPAC name**	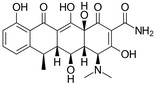 (4S,4aR,5S,5aR,6R,12aS)-4-(dimethylamino)-3,5,10,12,12a-pentahydroxy-6-methyl-1,11-dioxo-1,4,4a,5,5a,6,11,12a-octahydrotetracene-2-carboxamide	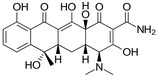 (4S,4aS,5aS,6S,12aS)-4-(dimethyloamino)-3,6,10,12,12a-pentahydroxy-6-methylo-1,11-dioxo-1,4,4a,5,5a,6,11,12a-oktahydrotetraceno-2-karboxyamid	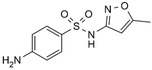 4-amino-N-(5-methyl-1,2-oxazol-3-yl) benzenesulfonamide	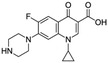 1-cyclopropyl-6-fluoro-4-oxo-7-piperazin-1-ylquinoline-3-carboxylic acid
Molecular weight (g·mol^−1^)	444.44	444.44	253.28	331.34
Water solubility at 25 °C (mg·L^−1^)	313	231	280	60
log K_ow_ *	−0.02	−1.25	0.89	0.28
pK_a_	3.0, 7.9, 9.2	3.3, 7.7, 9.7	1.7, 5.6	6.1, 8.7

* K_ow_—octanol–water partition coefficient.

**Table 2:** Synthesis parameters of mineral–carbon composites [16].

Composites	Mineral Carrier	Carbon Precursor	Fruit Pomace to Mineral Carrier Ratio	Pyrolysis Temperature in Nitrogen Atmosphere (°C)
CHS1a	HS (raw halloysite)— obtained from the “Dunino” strip mine in Legnica, Poland	raw fruit pomace	9:1	800
CHNT1a	HNT (halloysite nanotubes)— purchased from Merck KGaA, Darmstadt, Germany	raw fruit pomace	9:1	800
CKT1a	KT (kaolinite)— purchased from Sigma-Aldrich, Saint Louis, MO, USA	raw fruit pomace	9:1	800
CHS1b	HS—raw halloysite obtained from the “Dunino” strip mine in Legnica, Poland	raw fruit pomace	9:1	500
CHNT1b	HNT (halloysite nanotubes)— purchased from Merck KGaA, Darmstadt, Germany	raw fruit pomace	9:1	500
CKT1b	KT (kaolinite)— purchased from Sigma-Aldrich, Saint Louis, MO, USA	raw fruit pomace	9:1	500

**Table 3:** Structural parameters of HS, HNT, KT, and carbon–mineral composites [16].

Composites	S_BET_ m^2^/g	V_t_ cm^3^/g	V_mi_ cm^3^/g	V_me_ cm^3^/g	Mesoporosity %
HS	45.60	0.1925	0.0019	0.1906	99
HNT	53.65	0.2202	0.0016	0.2186	99
KT	8.93	0.0294	-	0.0294	100
CHS1a	71.38	0.1534	0.0134	0.1400	91
CHNT1a	77.01	0.1908	0.0149	0.1759	92
CKT1a	45.02	0.0434	0.0140	0.0294	68
CHS1b	56.78	0.1393	0.0092	0.1301	93
CHNT1b	74.89	0.1882	0.0103	0.1779	95
CKT1b	36.42	0.0402	0.0078	0.0324	81

S_BET_—specific surface area; V_t_—single-point total pore volume, calculated at p/p_o_ = 0.99; V_me_—mesopore volume, calculated by subtracting V_mi_ from V_t_; V_mi_—volume of micropores obtained with the α_s_-method; mesoporosity—the percentage of the volume of mesopores to the total pore volume.

**Table 4:** Removal efficiencies of selected antibiotics by HS, HNT, KT, and carbon–mineral composites [16,17,18].

Composites	Removal Efficiency (%)			
	Ciprofloxacin	Doxycycline	Sulfamethoxazole	Tetracycline
Initial pH				
	6.5	4.3	5.5	4.5
Dominant form of the antibiotic				
	zwitterion form	zwitterion form	neutral form	zwitterion form
HS	59.2 ± 2.1	75.0 ± 0.7	4.3 ± 0.5	67.4 ± 1.1
HNT	46.8 ± 0.6	80.6 ± 5.8	8.5 ± 0.2	83.7 ± 1.6
KT	50.9 ± 8.0	36.2 ± 2.2	6.3 ± 0.6	42.0 ± 0.4
CHS1a	67.5 ± 7.0	95.7 ± 0.1	65.4 ± 5.1	96.5 ± 0.4
CHNT1a	64.3 ± 5.2	89.3 ± 1.3	38.0 ± 2.1	92.9 ± 1.8
CKT1a	25.1 ± 0.4	51.2 ± 3.5	18.2 ± 1.6	42.0 ± 4.1
CHS1b	53.1 ± 2.3	89.7 ± 2.2	6.8 ± 1.9	76.4 ± 1.3
CHNT1b	53.8 ± 2.2	75.5 ± 4.0	16.1 ± 0.7	85.8 ± 2.5
CKT1b	28.9 ± 3.8	36.7 ± 3.3	11.5 ± 0.8	36.2 ± 0.5

**Table 5:** Equation of the London dispersion interactions and specific interactions using free adsorption energy (${∆G}_{a}$), free surface energy $\left(\gamma_{s}\right)$, and adhesion work $\left(W_{a}\right)$ in the Dorris, Gray, and Schultz methods and acidic–basic properties of solids [23,24,25,26,27].

Equation	Description	Interpretation
${∆G}_{a}={∆G}_{a}^{d}-RT\cdot ln(V_{N,n}^{\prime })+C$ ${∆G}_{a}^{d}$—dispersive component of adsorption free energy [J·mol^−1^], *R*—universal gas constant [J mol^−1^ K^−1^], *T*—column temperature [K], $V_{N,n}^{\prime }$—adjusted retention volume of n-alkane [cm^3^], *C*—constant value.	Free adsorption energy	Defines the free energy of adsorption $\left({∆G}_{a}\right)$ which comprises only the dispersive part
$W_{a}=2\sqrt{\gamma_{s}^{d}\cdot \gamma_{l}^{d}}$ $W_{a}$—dispersive component of work of adhesion [mJ], $\gamma_{s}^{d}$—dispersive component of surface energy [mJ·m^−2^], $\gamma_{l}^{d}$—dispersive energy of test molecule [mJ·m^−2^], $-∆G^{{CH}_{2}}=N\cdot a_{{CH}_{2}}\cdot W_{a}$, where $a_{{CH}_{2}}=1.09\cdot 10^{14}{\left(\frac{M}{\rho \cdot N_{A}}\right)}^{\frac{2}{3}}$ $∆G^{{CH}_{2}}$—dispersive component of methylene group of adsorption free energy [J·mol^−1^], a_CH2_—cross-section area of a CH_2_ group [m^2^], *M*—molar mass [g], $N_{A}$—Avogadro number, ρ—density [cm^3^ g^−1^].	Adhesion work Free adsorption energy of the methylene group and the cross-section area $a_{{CH}_{2}}$ of the alkane molecule	Describe the mutual interaction of the examined substance (adsorbent) with the test one [23]
$-RT\cdot ln\left(V_{N,n}^{\prime }\right)=2N\cdot a_{{CH}_{2}}\cdot \sqrt{\gamma_{s}^{d}\cdot \gamma_{l}^{d}}+C$	Transformed equation	Linear form of the equation enables determination of $\gamma_{s}^{d}$ value
$-RT\cdot ln\left(V_{N,n}^{\prime }\right)=f\left(a_{{CH}_{2}}\cdot \sqrt{\gamma_{l}^{d}}\right)$	Linear dependence diagram	The line slope enables quantitative determination of the stationary phase dispersive surface free energy, $\gamma_{s}^{d}$. The vertical distance on the axis $RT\cdot ln\left(V_{N,n}^{\prime }\right)$ from the n-alkane line to the point of polar substance is a component of specific adsorption energy ${∆G}_{a}^{sp}$ [23]
${∆G}_{a}=DN\cdot K_{a}+AN\cdot K_{b}$ *DN*—donor number [J·mol^−1^], AN—acceptor number [%], $K_{a}$—acidic characteristic of solid $K_{b}$—basic characteristic of solid	Modified Gutmann equation	Donor number *DN*, given in kJ/mol is described as negative enthalpy of forming an addition compound of a given base (donor) with Lewis acid SbCl_5_ (acceptor) in a 10^−3^ M solution of 1,2-dichloroethane. Acceptor number *AN* is, in turn, defined as a non-dimensional number corresponding to spectrum diffraction NMR^3I^ for triethylphosphine oxide (standard donor) diluted in the tested acceptor. It is the number given in non-dimensional conventional units (hexane = 0; 1,2-dichloroethane = 100).
$\frac{{∆G}_{a}}{AN}=\frac{DN}{AN}\cdot K_{a}+K_{b}$	Transformed equation	The ratio $\frac{K_{a}}{K_{b}}$ facilitates specifying the character of the test surface. If the ratio is $\frac{K_{a}}{K_{b}}<1$, then the surface is basic (donor properties prevail over the acceptor ones). If the ratio is $\frac{K_{a}}{K_{b}}>1$, then the surface is acidic. (acceptor properties prevail over donor the ones). If $\frac{K_{a}}{K_{a}}\approx 1$, then the surface is amphotheric

**Table 6:** Carbon concentration of carbon–mineral composites.

Composites	Carbon Concentration (%)
CHS1a	79.4 ± 0.0
CHNT1a	84.8 ± 0.4
CKT1a	85.7 ± 0.1
CHS1b	79.0 ± 0.2
CHNT1b	76.1 ± 0.4
CKT1b	79.1 ± 0.2

**Table 7:** Dispersive component of surface free energy and acid–base properties of HS, HNT, KT, and carbon–mineral composites.

Composites	Dispersive Component of Surface Energy $\gamma_{s}^{d}$ (mJ·m^−2^)	R^2^	Acidic Characteristic of Solid *K_a_*	Basic Characteristic of Solid *K_b_*	Ratio *K_a_*/*K_b_*	R^2^
HS	62.9	0.9821	0.179	0.170	1.05	0.9404
HNT	42.4	0.9992	0.211	0.222	0.95	0.8981
KT	28.4	0.9995	0.251	0.418	0.60	0.9629
CHS1a	252	0.9998	0.177	0.126	1.40	0.9703
CHNT1a	46.8	0.9971	0.116	0.035	3.29	0.9259
CKT1a	46.9	0.9994	0.398	1.029	0.38	0.8547
CHS1b	38.7	0.9897	0.200	0.085	2.34	0.9916
CHNT1b	35.3	0.9967	0.093	0.101	0.91	0.9784
CKT1b	32.5	0.9924	0.281	0.401	0.70	0.9857

## Data Availability

The original contributions presented in this study are included in the article. Further inquiries can be directed to the corresponding author.
